# Can fecal characteristics be used to predict the digestibility of certain macro minerals in dry and lactating cows?

**DOI:** 10.1111/asj.70017

**Published:** 2025-01-13

**Authors:** Emre Yilmaz, Soner Uysal

**Affiliations:** ^1^ Department of Animal Nutrition and Nutritional Diseases, Faculty of Veterinary Medicine Ataturk University Erzurum Turkiye

**Keywords:** calcium, cow, digestibility, fecal characteristics

## Abstract

This study aims to evaluate the relationship between mineral digestibility and fecal characteristics and compare digestibility in dry and late‐lactating cows. A total of 107 multiparous Holstein and Simmental cows were included, with 66 cows in late lactation and 41 cows in the dry period. The apparent digestibility of key macro minerals, dry matter content in feces, dirtiness scores, fecal characteristics, and serum macro mineral levels were determined. Cows consuming the same diet were arranged according to a completely randomized design. Results showed that, compared to the late lactation group, the dry period group had a significantly lower total dirtiness score, higher phosphorus digestibility, and elevated serum calcium levels (*p* < 0.05). Additionally, fecal consistency and fecal height were greater in the dry period group (*p* < 0.05). However, the increased phosphorus digestibility observed during the dry period was not significantly associated with fecal consistency (*r* = +0.225, *p* < 0.05) or the total contamination score (*r* = −0.339, *p* < 0.05). Consequently, this study reveals that understanding the differences in mineral digestibility between different physiological stages can enhance nutritional approaches for better dairy cow management.

## 1 INTRODUCTION

Minerals are chemicals that are required in lower amounts than energy and protein, but play vital roles in the body (Princewill et al., 2015). They are classified as macro, micro, or trace based on the amount they are found in the body. Calcium (Ca), phosphate (P), and potassium (K) are essential macro minerals, and they, along with carbon, hydrogen, oxygen, and nitrogen, constitute more than 99% of living beings (Karagül et al., 2000). Macro minerals play important roles such as muscle contraction, electrolyte homeostasis, and enzyme structure formation (Princewill et al., 2015). Thus, dairy cows need certain proportions of minerals in addition to organic matter (protein, fat, carbohydrate, and vitamin) in their diets to maintain good health, proper fetal growth, and an adequate production of milk. Quiroz‐Rocha et al. (2009) noted that the levels of certain minerals, such as Ca and P, in the blood were statistically different during the pre‐ and post‐partum periods. It is well established that as pregnancy progresses, the maternal demand for Ca increases, coinciding with the mineralization of the fetal skeleton. Due to the increased requirements for fetal skeletal mineralization in late pregnancy, the Ca needs of all mammals rise significantly. Comar (1956) reported that in rats, the maternal Ca requirement increased from less than 0.5 mg/day in early pregnancy to approximately 12 mg per fetus by the 17th day of pregnancy, continuing to increase until parturition.

The determination of mineral digestibility often requires costly in vivo studies or laboratory analyses using advanced equipment, rendering access to such methods unavailable for everyone (Zewdie, 2019). Therefore, an easier approach for estimating/detection of mineral digestibility is required. The digestibility of minerals, similar to other nutrients, is contingent upon physiological processes within the body. During the study conducted with pigs (70th and 100th days of pregnancy and second week of lactation), it was found that Ca digestibility increased as pregnancy progressed, while the digestibility of P decreased. Similarly, when comparing pre and post lactation periods, Ca and P digestibility increased, whereas the digestibility K decreased (Jongbloed et al., 2013). Studies have demonstrated that, even within the same period, the lactation phase or pregnancy status may elicit varying effects on mineral digestibility (Gaignon et al., 2019; Jongbloed et al., 2013). Indeed, there is still much to learn about the digestibility of macro minerals throughout the late lactation (LL) and dry periods (DPs) in dairy cows.

Fecal characteristics can reveal information about the overall health of cow as well as the amount of rumen fermentation and digestive function (Zaaijer & Noordhuizen, 2003). For example, a significant percentage of incompletely formed and soft fecal piles within a herd, dirtiness near the perineum, the presence of undigested particles, and elevated pH levels are indicative of ruminal acidosis (Örtlek et al., 2018). Conversely, the presence of firm and constipated feces may indicate a reduction in nutrient fermentation in the rumen and lower digestive tract, potentially suggesting reduced colonic fermentation (Lean et al., 2014). Feces provide valuable insights into nutrient digestion, raising questions about their relationship to mineral digestion. The mineral digestibility of dairy cows during the LL and DPs, as well as the fecal characteristics and mineral digestibility during these times, is not well understood. This study aims to compare the mineral digestibility in dry and lactating cows fed similar diet and to investigate the correlation between certain macro mineral digestibility and the fecal characteristics.

## 2 MATERIAL AND METHODS

The research was carried out at a dairy farm in Erzurum, Turkiye, in December 2023, with approval obtained from the Ataturk University, Faculty of Veterinary Medicine Local Ethics Committee on April 1, 2023 (Approval no: E‐75296309‐050.01.04‐2300106037).

### 2.1 Animals, diets, and experimental design

The research was conducted on 107 multipara Holstein and Simmental dairy cow, with 66 in LL (with at least 150 days in milk and an average milk yield of 10 ± 2 L) and 41 in the DP (prepartum at least 30 days). The experiment was arranged in a completely randomized design. All animals were housed in paddocks and were divided into two groups for the experiment as the LL and the DP group. Because the LL and DL groups on the farm were already consuming the diet described in Table 1, no adaptation period was implemented prior to the study. Both experimental groups were provided a diet with the same composition (Table 1). The diet utilized in the study was not formulated by the research team; rather, it was the standard diet already in use by herd manager of the dairy farm. Animals were provided with ad libitum the feed and water throughout the study. In this study, the dry matter intake (DMI) of the cows was measured daily to ensure accuracy in assessing their nutritional intake during the experimental period.

**TABLE 1:** Ingredients, nutrient composition, and particle size of experimental diet.

Ingredients, % DM	Amount (kg/day)
Maize silage	5.10
Dry meadow grass	9.90
Concentrated feed^1^	3.00
Premix^2^	0.01

Nutrient composition	Percentage (%)
DM, %	69.1
CP, % DM	13.1
ADF, % DM	33.2
NDF, % DM	56.4
ME, Mcal/kg DM^2^	2.31
Ash, % DM	11.1
Ca, % DM	0.62
P, % DM	0.33
K, % DM	1.45

Particle size	Percentage (%)
Top sieve	24.3
Median sieve	32.7
Lower sieve	24.1
Bottom sieve	16.9

^1^ Ingredients of concentrated feed (respectively): soybean meal (21.1%), distiller dried grains with solubles (corn, 29.5), barley grain (9.6%), corn grain (19.8%), corn gluten meal (15.2%), molasses (10.1% CP), calcium carbonate (0.8%), sodium chloride (1%), and vinas (2.5%).

^2^ Premix additive (per kg of DM) contains 15 mg Cu, 40 mg Zn, 20 mg Mn, 0.35 mg I, 0.20 mg Se, 0.15 mg Co, 3,000 IU vitamin A, 700 IU vitamin D, and 10 IU vitamin E. ^**2**^ME value was determined using the formulas provided by NRC (2001).

Abbreviations: ADF, acid detergent fiber; Ca, calcium; CP, crude protein; DM, dry matter; K, potassium; NDF, neutral detergent fiber; P, phosphorus.

### 2.2 Sample collection and visual assessment

The dirtiness score of the perineal region in dairy cows was determined using the method suggested by Petrovski et al. (2022). According to this procedure, each animal was scored using a 5‐point scale (1: cleanest, 5: dirtiest) by three researchers. Feed samples were collected in a large plastic bucket from the upper, middle, and lower parts of different regions of the fresh feed that was just poured in front of the animals in the morning feeding (from 10 different regions for 5 days), and diagonal ones were taken from the quarter slices by mixing homogeneously on a clean surface (Peters, 2023; Taş, 2018). The samples were packed in airtight plastic bags, compressed as much as possible (in order to reduce the air inside), and transported to the laboratory under cold chain conditions. The obtained samples were kept in a deep freezer at −20°C until laboratory analysis.

Fecal and blood samples were collected according to a standard procedure commonly employed in farms for evaluating the overall herd, whereby it is considered sufficient to sample approximately 10% of the herd (Peters, 2023; Taş, 2018). To ensure accuracy, the cows from which fecal samples were collected were marked, and blood samples were subsequently drawn from these same animals. Animals were observed for approximately an hour, on average 3–4 h after their morning feeding. Freshly dropped feces from these animals were collected from their medium‐clean areas (not contaminated with urine, for 5 days (Taş, 2018). Fresh feces that fell on the ground were promptly photographed to assess their color, consistency, spread area, and height. The spreading area and height values of the feces were determined from the photographs using the Image J (Java image processing program) software. Fecal consistency was evaluated using a 5‐point scale, while color criteria of feces were assessed on a 3‐point scale (Hutjens, 2002; Ireland‐Perry & Stallings, 1993; Taş, 2018).

Blood samples were collected from randomly selected dairy cows into 8 mL serum tubes for biochemical analysis. The blood was immediately centrifuged at 3000 rpm at +4°C to obtain serum and then were then stored at −20°C.

### 2.3 Determination of particle size of feed and feces

The particle size of feces and feed was determined using particle separators with three sieves each (Cotanch & Darrah, 2012; Heinrichs, 2013). A feed sample (200 g) was placed on the top sieve. The sieving procedure involved 10 horizontal, 17‐cm movements perpendicular to each side of the box, with a total of 40 movements. The fecal samples (200 g) were collected and were washed in a large white bucket under a non‐pressurized tap for approximately 5 min until the water ran clear. The remaining material on the sieves was then weighed (Taş, 2018).

### 2.4 Chemical analysis in feed and feces samples

The nutrients dry matter (DM), crude protein (CP), acid detergent fiber (ADF), neutral detergent fiber (NDF), and ash in feed samples and also DM in feces samples were determined according to the analysis methods specified in AOAC (1990) and Van Soest et al. (1991).

### 2.5 Determination of mineral contents in blood serum, feed, and feces samples

A two‐step procedure was used to determine the mineral content of the samples. Initially, the samples were incinerated using the closed system microwave incineration method. Approximately 2–5 g of sample with a precision of 1 mg was placed in a Teflon container, to which 4 mL of ultra‐pure nitric acid (65% purity) and 1 mL of hydrogen peroxide (30% purity) were added. After a 5‐min incubation period, the containers were sealed and subjected to microwave incineration following a prescribed program (10 min at 130°C, 5 min at 150°C, and 10 min at 180°C). Subsequently, the solutions were diluted to 50 mL with ultra‐distilled water and filtered (25/0.45 μm). The resulting filtrate was utilized for analysis. Mineral values (Ca, P, and K) in the collected samples were determined using an Agilent 7800 Inductively Coupled Plasma‐Mass Spectrometry (ICP‐MS) device (Giannenas et al., 2009). Ca, P, and K levels of the blood serum samples were analyzed using a Beckman Access 2 Immunoassay System (Beckman Coulter Inc., USA) autoanalyzer.

### 2.6 Determination of apparent digestibility of DM and certain macro mineral

Digestibility of DM and certain macro mineral (Ca, P, and K) were determined using the method reported by Cheng and Coon (1990) using ash insoluble in hydrochloric acid as a marker. The apparent digestibility of different nutrients was calculated by using the following equation (Van Keulen & Young, 1977):

$$\text{Apparent digestibility}=100-\left(\frac{I\left(F\right)*N\left(E\right)}{I\left(E\right)*N\left(F\right)}\right)*100$$

I (F) = concentration of indicator in feedstuff
I (E) = concentration of indicator in feces
N (F) = concentration of nutrients in feedstuff
N (E) = concentration of nutrients in feces

### 2.7 Statistical analysis

Fecal samples were collected using a simple random sampling technique. The research data were evaluated using the SPPS 20 statistical package program. The results were expressed as mean ± SEM (standard error of the mean). Independent samples *t* test analysis was used to compare groups in apparent digestibility of DM and certain macro minerals, body dirtiness scores, fecal characteristics, fecal particle size, and serum levels of certain macro minerals. Subsequently, Pearson correlation analysis was conducted to examine the influence of fecal score and total contamination score values on the digestibility of P mineral. A significance level of *p* < 0.05 was accepted for the all tests.

## 3 RESULTS

The apparent digestibility of DM and certain macro minerals (Ca, P, and K) in dairy cows during the LL and DP are presented in Table 2. No significant difference was observed in DMI, DM, and Ca, digestibility between the LL and DP groups (*p* > 0.05; *p* = 0.079; *p* = 0.055, respectively). P digestibility was significantly higher in the DP group compared to the LL group (*p* < 0.05), whereas no significant difference was found in K digestibility (*p* > 0.05).

**TABLE 2:** Apparent digestibility of certain macro minerals and dry matter contents in dairy cows in dry and late lactation periods (mean ± SEM).

Group	N	DMI	Apparent digestibility (%)			
			DM	Ca	P	K
LL	66	12.1 ± 1.2	64.21 ± 1.04	41.67 ± 0.521	34.99 ± 0.551	63.27 ± 1.02
DP	41	11.8 ± 1.8	66.59 ± 0.71	43.87 ± 0.923	37.49 ± 0.810	62.91 ± 1.11

			Independent sample t test			
*p*‐value		0.677	0.079	0.055	0.022	0.815

Abbreviations: Ca, calcium; DM, dry matter; DP, dry period group; K, potassium; LL, late lactation period group; P, phosphorus; SEM, standard error of mean.

The body dirtiness scores and fecal characteristics of the cows are summarized in Table 3. Total body dirtiness score was significantly higher in the LL group compared to the DL group (*p* < 0.05). Fecal consistency was also significantly different between the two groups, with the DP group showing higher scores (*p* < 0.05). Fecal height was significantly greater in the DP group (*p* < 0.05), while there were no significant differences in fecal DM (%) or spread area. Fecal particle size analysis revealed that cows in the DP group had a significantly lower percentage of particles in the median sieve (*p* < 0.001) and a significantly higher percentage in the bottom sieve (*p* < 0.001).

**TABLE 3:** Body dirtiness score, fecal characteristics, and fecal particle size in dairy cows in late lactation and dry period (mean ± SEM).

Parameters	Groups		*t* test
	LL	DP	*p*
Body dirtiness scores			
Perineal score	3.00 ± 0.149	2.70 ± 0.153	0.177
Udder	2.60 ± 0.143	2.00 ± 0.365	0.159
Belly	3.10 ± 0.223	2.20 ± 0.389	0.066
Hind leg	3.40 ± 0.163	3.20 ± 0.133	0.356
Hind quarter	3.10 ± 0.180	2.30 ± 0.335	0.054
Hind foot	3.70 ± 0.153	3.00 ± 0.298	0.055
Total dirtiness score	3.14 ± 0.900	2.57 ± 0.071	0.017
Fecal characteristics			
Dry matter, %	13.8 ± 0.087	14.6 ± 0.344	0.149
Color	2.20 ± 0.133	2.20 ± 0.133	0.999
Consistency	2.50 ± 0.167	3.20 ± 0.249	0.033
Spread area, cm^2^	124 ± 4.42	118 ± 5.13	0.359
Height, cm	3.90 ± 0.365	5.06 ± 0.115	0.012
Fecal particle size			
Top sieve	19.9 ± 1.06	17.4 ± 1.20	0.137
Median sieve	33.6 ± 0.636	28.2 ± 0.952	<0.001
Lower sieve	17.9 ± 0.737	17.6 ± 0.897	0.799
Bottom sieve	28.7 ± 1.25	36.8 ± 1.13	<0.001

Abbreviations: DP, dry period group; LL, late lactation group.

Serum macro mineral levels are shown in Table 4. Serum Ca levels were significantly higher in the DP group compared to the LL group (*p* < 0.05). No significant differences were observed in serum P or K levels between the two groups (*p* > 0.05 and *p* = 0.521, respectively). A weak positive correlation was found between P digestibility and fecal consistency (*r* = 0.225), while a weak negative correlation was observed between P digestibility and total dirtiness score (*r* = −0.339). However, neither of these correlations reached statistical significance (*p* > 0.05; Table 5).

**TABLE 4:** Certain macro mineral levels in the serum of dairy cows in late lactation and dry period (mean ± SEM).

Group	*N*	Ca, mg/dL	P, mg/dL	K, mmol/lt
LL	66	8.1 ± 0.209	5.8 ± 0.211	4.4 ± 0.169
DP	41	8.9 ± 0.243	5.5 ± 0.221	4.5 ± 0.136

		Independent samples *t* test		
*p*‐value		0.028	0.303	0.521

Abbreviations: Ca, calcium; DP, dry period group; K, potassium; LL, late lactation group; P, phosphorus; SEM, standard error of mean.

**TABLE 5:** Correlation coefficients among certain macro mineral digestibility, total dirtiness score, and fecal consistency.

	Apparent digestibility of minerals
Fecal characteristics	P (%)
Consistency	+0.225^1^
Total dirtiness score	−0.339^2^

No. of herds = 107. There is no linear relationship between parameters (Pearson correlation, p_1_ = 0,341; p_2_ = 0,144). P, phosphorus.

## 4 DISCUSSION

Consistency and other fecal characteristics have been studied for many years because they provide valuable information about how cows digest their nutrients (Ireland‐Perry & Stallings, 1993). Thus, numerous studies have focused on determining fecal characteristics and nutrient digestibility in cow during various stages of lactation (Danscher et al., 2015; Ireland‐Perry & Stallings, 1993; Renaud et al., 2020). Some studies suggest that nutrient digestibility is high at the beginning of the lactation period, while others argue that it is low due to the metabolic load on the animal (Aikman et al., 2008; Daniel et al., 2022; Plaizier et al., 2018). However, it is evident that nutrient digestibility fluctuates across different periods (Aikman et al., 2008; Daniel et al., 2022). Moreover, nutrient digestibility is influenced by numerous factors, dietary composition probably being a particularly important one (Gaignon et al., 2019; Zewdie, 2019). Therefore, in this study, animals in both LL and the DP were fed the same diet. Feeding the same diet reduced the covariance effect of the diet on nutrient digestibility. In the study, the total digestibility of certain macro minerals (Ca, P, and K) was compared between the LL period and the DP. Several studies have reported results consistent with our findings (Jeong et al., 2018; Tucker et al., 1992). The increase in the digestibility of P during the DP compared to the LL period can be attributed to several physiological and metabolic factors, even when both groups of cows are fed the same diet. One potential reason is the stabilization of ruminal pH and fermentation processes during the DP (Qiu et al., 2021). In lactating cows, milk production can lead to increased lactic acid production, resulting in a drop in rumen pH, which negatively impacts mineral absorption. Conversely, during the DP, the absence of milk production allows for a more balanced ruminal environment, thereby enhancing the absorption of P (Møller et al., 1997). Additionally, changes in microbial activity within the rumen may play a significant role. The composition of the microbial population can shift during the DP, potentially leading to an increase in the production of enzymes that facilitate mineral absorption (Martens et al., 2012). Furthermore, metabolic adjustments during the DP enable cows to optimize their mineral utilization, as they require less energy and nutrients than during LL (Gerloff, 1988; Overton & Waldron, 2004). However, the decrease in milk production during the DP may cause some hormonal and metabolic changes that are still being investigated. The physiological differences between the two periods are also noteworthy. In LL, the heightened demand for minerals due to milk production may result in deficiencies that affect their digestibility (Bernier‐Dodier et al., 2011; Holtenius et al., 2003). Conversely, in the DP, cows are less stressed, allowing for a more consistent and adequate intake of nutrients, which can enhance overall digestibility (Gundelach et al., 2020).

In the present study, serum Ca levels increased in dairy cows during the DP. The increased requirement for Ca (which has a metabolic correlation with P) during the transition period in dairy cows is not solely attributed to improved milk production (de Sousa et al., 2018; Tamim & Angel, 2003). During this time, the placenta transfers a substantial amount of Ca to the calf to meet fetal needs the period when the majority of the offspring's growth occurs, in addition to producing colostrum during (de Sousa et al., 2018; Wathes, 2022). The serum Ca level is lower in the LL group because they maintain to produce milk, even if to a lesser extent, during this period. It is a well‐known phenomenon that as milk yield increases, the Ca level in the blood decreases (Chapinal et al., 2012). Another reason for the high serum Ca levels during the DP, as observed in this study, is the enhancement of Ca and P digestion (Chapinal et al., 2012; Venjakob et al., 2018). One of the hypotheses of this study was to establish the collective relationship between mineral digestibility, fecal consistency, total contamination score, and the digestibility of crucial macro minerals. However, the study found no significant association between Ca and P minerals and these parameters.

In this study, it was found that the LL group had a higher total contamination score, lower fecal height, and lower fecal consistency. As is well known, fecal characteristics and contamination status provide important information regarding nutrient digestion (Hughes, 2001). Even when the same diet is consumed, an increase in fecal consistency may be observed during the LL period, and this observed increase can be attributed to several factors (Ireland‐Perry & Stallings, 1993). The LL period is characterized by significant metabolic changes in cows, which can influence fecal consistency. The continued presence of milk production during this period can lead to alterations in energy and nutrient demands. Additionally, stress levels associated with milk production are known to adversely affect digestive efficiency (Daniel et al., 2022; Ireland‐Perry & Stallings, 1993; Putman et al., 2018). However, as cows transition to the DP, milk production ceases completely, allowing for a reduced metabolic load and consequently decreasing the pressure on the digestive system. This can lead to improved nutrient utilization (Overton & Waldron, 2004). It is thought that the decrease in nutrient utilization during the LL period is an indicator that could lead to changes in intestinal viscosity (Hawken et al., 2012). Furthermore, various metabolic processes may also impact the passage time. Hormonal fluctuations during LL, particularly elevated levels of insulin and cortisol, can affect intestinal motility. This increased motility may result in a quicker transit of feces through the digestive tract, further influencing fecal consistency. Stress is also a significant factor affecting intestinal motility, and some level of stress may occur during the LL period due to milk production demands. Stress can accelerate intestinal movements, contributing to a more liquid fecal density (Wu et al., 2013).

Additionally, the microbial population within the rumen can also impact fecal density. For example, the synthesis of lactic acid and/or fatty acids is necessary for milk production, which can promote the proliferation of certain microorganisms while reducing the quantity of others (Chanthakhoun et al., 2012; Lean et al., 2014; Martens et al., 2012). Even though both groups consume the same diet, the microbial changes in the gastrointestinal systems of cows during LL can significantly affect fecal consistency. As the passage rate in the digestive tract increases, the viscosity of the digested material decreases, resulting in a more fluid fecal consistency. Similarly, when nutrients pass through the digestive system at a faster rate, digestibility may decrease because digestive enzymes do not have sufficient time to effectively interact with the nutrients (Yang et al., 1999).

Although this study demonstrated similar DM digestibility for both groups, mineral digestibility, particularly that of P, may be influenced by overall nutrient dynamics. While P is generally consumed in small amounts, rapid transit may not allow adequate time for effective mineral absorption. Therefore, examining the digestibility of other nutrients—particularly proteins and fats—would be important for a better understanding of digestive efficiency (Sarwar et al., 1992; Weiss et al., 2009). Previous studies have indicated a high correlation between DM digestibility and mineral digestibility in lactating cows (Khorasani et al., 1997). Unfortunately, this study did not address the digestibility of other nutrients.

The higher the fluidity of digesta, the increased possibility of dirtiness to the animal and thus a wider spread area upon defecation when it falls to the ground (Hughes, 2001). On the other hand, an elevation in the proportion of small‐sized particles in feces suggests thorough digestion of the feed (Castillo‐Lopez et al., 2020). In this study, it was noted that the proportion of small and medium‐sized particles decreased in the DP group, accompanied by an increase in the proportion of smaller particles. These findings are consistent with those of other researchers and support results of present study (Haselmann et al., 2019; Maulfair et al., 2011; Yang et al., 2002). Specifically, the heightened viscosity of the digestive system or certain enhancements in the rumen may contribute to improved nutrient digestion. Some researchers have suggested that certain particles in the rumen may be selectively retained (Sutherland et al., 1988), which could increase the exposure time to enzymes (Poppi et al., 1985). This process leads to enhanced dissolution of the feed, resulting in a decrease in particle size in feces or, in other words, an increase in the proportion of smaller particles.

Consequently, this study observed that phosphorus digestibility increased during the DP in dairy cows compared to the LL period. Calcium levels were also significantly higher in the DP, reflecting the increased mineral demand at this stage. The LL group had higher contamination scores and lower fecal height and consistency than the DP group, highlighting the impact of LL's physiological changes on nutrient digestion and health. Interestingly, fecal consistency and dirtiness scores did not significantly affect mineral digestibility as initially expected. These findings suggest that while the LL brings specific metabolic changes impacting nutrient utilization, the DP provides a more stable environment for mineral absorption. Factors like hormonal shifts, reduced metabolic load, and microbial changes during the DP may enhance mineral digestibility. Although fecal characteristics may indicate macro mineral digestibility, this study found limited evidence to confirm this. Further research is recommended to explore this relationship across different lactation stages, feeding methods, and diets to improve dairy nutrition and mineral management strategies.

## CONFLICT OF INTEREST STATEMENT

The authors declare no conflicts of interest.
